# scGT:integration algorithm for single-cell RNA-seq and ATAC-seq based on graph transformer

**DOI:** 10.1093/bioinformatics/btaf357

**Published:** 2025-06-24

**Authors:** Yunjing Qi, Yulong Kan, Jing Qi, Shuilin Jin

**Affiliations:** School of Mathematics, Harbin Institute of Technology, Harbin 150000, China; School of Mathematics, Harbin Institute of Technology, Harbin 150000, China; School of Mathematics, Harbin Institute of Technology, Harbin 150000, China; Zhengzhou Research Institute, Harbin Institute of Technology, Zhengzhou 450000, China; School of Mathematics, Harbin Institute of Technology, Harbin 150000, China; Zhengzhou Research Institute, Harbin Institute of Technology, Zhengzhou 450000, China

## Abstract

**Motivation:**

Multi-omics analysis of individual cells offers remarkable opportunities for exploring the dynamics and relationships of gene regulatory states across large atlas data. However, the current integration algorithms have limited performance, largely due to ignoring the impact of correlation features within the dataset on the discrepancies between omics.

**Results:**

In this study, we propose scGT, a model based on Graph Transformer for single-cell RNA-seq and ATAC-seq data, which leverages the robust graph structures strengthened by correlation features present in each raw dataset to harmonize representations of multi-omics data, enabling the integration of multi-omics and effective label transfer. We compare scGT with other state-of-the-art methods on paired and unpaired datasets. The results show that scGT accomplishes more accurate label transfer and is capable of integrating datasets with millions of cells. Meanwhile, scGT achieves better performance for preserving biological variation during integration.

**Availability and implementation:**

The source code and data used in this article can be found at https://github.com/Jinsl-lab/scGT.

## 1 Introduction

Single-cell sequencing technologies such as transcriptome (scRNA-seq) (Picelli *et al.* 2013, Zheng *et al.* 2017), chromatin accessibility (scATAC-seq) (Berger 2007, Klemm *et al.* 2019), have enabled a comprehensive understanding of cell differences from a molecular perspective (Kalisky *et al.* 2018). This advancement has facilitated in-depth exploration of cellular heterogeneity and biological processes, providing valuable insights in neuroscience, cancer biology, immuno-oncology, and therapeutic responses and beyond (Ma *et al.* 2020a, Zhang *et al.* 2025, Dimitrakopoulos *et al.* 2018). Single-cell RNA sequencing analyzes transcriptomic information within individual cells to reveal gene expression levels, enhance our understanding of cellular heterogeneity and functional diversity, and promote the foundation of novel cell types. Single-cell ATAC sequencing is an epigenomic analysis technique that investigates the physical structure of the genome by identifying open chromatin regions, providing insights into the regulatory mechanisms underlying cellular identity, which is a very important complement for scRNA-seq. However, the sparsity and high dimensionality of the peak matrix and the lack of easily interpretable gene marker information in scATAC-seq data present challenges for cell type identification (Pott and Lieb 2015). In contrast, there is a large number of well-annotated scRNA-seq datasets (Tabula Muris Consortium *et al.* 2018, Cao *et al.* 2020). By integrating scRNA-seq data with scATAC-seq data, we are able to annotate scATAC-seq data better through scRNA-seq data. It is crucial to link epigenetic regulation to gene products, improving the annotations of scATAC-seq data and providing more detailed molecular insights.

So far, many methods have been developed to integrate scRNA-seq and scATAC-seq data. In the case of paired single-cell data integration (Cao *et al.* 2018, Chen *et al.* 2019), methods like scAI (Jin *et al.* 2020) and Mofa+ (Argelaguet *et al.* 2020) achieve multi-omics integration through matrix decomposition and factorization, respectively. However, paired sequencings are both costly and technically demanding. More generally, many methods have been proposed for integrating scRNA-seq and scATAC-seq data from different cells. Commonly used methods include Seurat (Stuart *et al.* 2019), which employs CCA and MMN to identify anchors for correcting heterogeneity between datasets. Conos (Barkas *et al.* 2019) constructs a weighted union graph by identifying aligned sample pairs. scGCN (Song *et al.* 2021) builds a hybrid graph using CCA and MNN to achieve label transfer across omics, species, and platforms based on graph convolutional networks. GLUE (Cao and Gao 2022) constructs a shared low-dimensional space for different omics using autoencoders and guidance graphs. scJoint (Lin *et al.* 2022) employs a semi-supervised neural network framework to align different omics in a low-dimensional space. scBridge (Li *et al.* 2023) leverages the heterogeneity among different omics to gradually reduce the differences. Although various methods have achieved good results in different fields, they do not effectively leverage the correlation features inherent within the dataset, ignoring the ancillary role of correlation features in the same dataset in eliminating multi-omics differences.

Here, we propose scGT, a Graph Transformer-based framework for integration and label transfer of single-cell multi-omics datasets. Specifically, to design a model suitable for single-cell multi-omics data integration tasks, we added three innovations. First, we utilized the original data to construct a hybrid graph and refined inter-dataset graph connections with high-accuracy intra-dataset graph connections, thereby enhancing the quality of the hybrid graph. Second, we equipped a self-attention mechanism based on the constructed hybrid graph to achieve both global graph-level and local edge-level information flow, which allows the model to perform information propagation independently of the graph structure, mitigating the impact of erroneous or incomplete connections in the hybrid graph. Third, we designed a composite loss. All of these enable the model to achieve robust performance in multi-omics integration and label transfer.

We evaluated the integration effectiveness of scGT on five multi-omics datasets, focusing on the quality of joint embedding and the accuracy of label transfer. The results show that scGT effectively integrates multi-omics data and achieves high-precision label prediction for scATAC-seq data. Moreover, scGT effectively handles datasets with unmatched cell types and is capable of identifying unknown cell types. Notably, scGT successfully integrates complex atlas data and performs well even when the number of cells in the datasets is small.

## 2 Materials and methods

### 2.1 Overview

**Figure 1.**
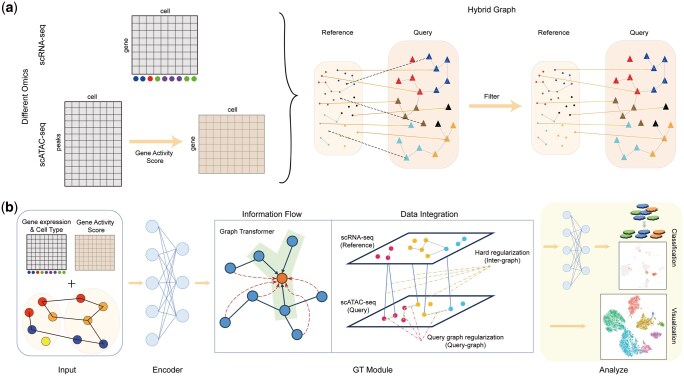
Overview of scGT. (a) scGT takes two inputs: the gene expression matrix from labeled scRNA-seq data and the gene activity matrix from unlabeled scATAC-seq data. An initial hybrid graph is constructed from the original data, including intra-dataset and inter-dataset connections. Then, inter-dataset connections are filtered using high-accuracy intra-dataset connections to generate a better hybrid graph. (b) scGT leverages the input data and the hybrid graph to enable global graph-level and local edge-level information flow based on Graph Transformer. Using cross-entropy loss, hard regularization loss built from inter-dataset connections, and query graph regularization built from the intra-graph connections within the query dataset (scATAC-seq) promotes data integration and label transfer.

scGT is a semi-supervised method developed for training labeled reference data (scRNA-seq) and unlabeled query data (scATAC-seq) together. Specifically, it constructs a robust hybrid graph from the original data that includes inter-dataset and intra-dataset connections. It employs a Graph Transformer-based framework to integrate labeled scRNA-seq data and unlabeled scATAC-seq data. scGT is divided into two main steps. Step 1 first constructed the initial hybrid graph based on the original data and filtered the connections between the two datasets using the high-accuracy intra-dataset connections, which significantly improved the connection quality of the hybrid graph (Fig. 1a). In Step 2, we constructed a network that includes the encoder, GT module, and classifier. The encoder projects the data into a low-dimensional space. In the GT module, we introduce the concept of Graph Transformer to enable both local information flow along edges and global information flow across the entire graph. This module’s output supports joint visualization. Then, using the classifier for label prediction. These components work together to enable the integration and label transfer of multi-omics data. The training process of the network is guided by a combination of cross-entropy loss, hard regularization loss which is designed based on inter-dataset graph connections, and query graph regularization which is designed based on graph connections within the query data, resulting in label predictions with confidence scores and joint embedding representations (Fig. 1b). At the same time, we propose an optional strategy. When the confidence score of a cell assigned to a type exceeds 0.95, the cell is classified into that category and included in the cross-entropy loss calculation. This approach enhances data integration and label transfer. Overall, scGT constructs a robust hybrid graph and achieves multi-omics data integration and label transfer through the Graph Transformer framework. Subsequently, we compare scGT with other state-of-the-art multi-omics integration algorithms.

### 2.2 Model

#### 2.2.1 Data preprocessing

For each pair of datasets, we denote the scRNA-seq data with known cell labels as the reference data and the scATAC-seq data, which needs to be annotated, as the query data. The cell sets of reference data and query data are denoted as $B^{R}$ and $B^{Q}$. And we extract their common genes, so $X^{R}\in R^{m\times n_{R}}$, $X^{Q}\in R^{m\times n_{Q}}$, which are the original input of scGT, where m is the number of common genes, and $n_{R}$, $n_{Q}$ are the cell counts of reference data and query data.

#### 2.2.2 Hybrid graph construction

First, we use the LogNormalize method to normalize the data. And then we perform the standardized transformation. For reference data $X^{R}$, i.e.


(1) $$x_{ij}=\frac{x_{ij}-\mu_{i}}{\theta_{i}}$$

where $x_{ij}\in X^{R}$, $\theta_{i}$ is the standard deviation and $\mu_{i}$ is the mean. After standardization, the reference data is represented as ${\tilde{X}}^{R}$, and the query data are represented as ${\tilde{X}}^{Q}$ similarly.

Then, we will construct the initial hybrid graph based on mutual nearest neighbor (MNN) (Haghverdi *et al.* 2018) and reciprocal principal component analysis (RPCA) (Stuart *et al.* 2019) or principal component analysis (PCA) (Hotelling 1933). We will then use our method to filter the inter-dataset graph connections through the intra-dataset graph connections, getting the final hybrid graph.

#### 2.2.3 Inter-dataset graph connections

To enable fast and accurate construction of inter-dataset connections, we use RPCA to dimensionality reduce the reference data and the query data to the same space. Specifically, it projects each dataset into the PCA space of the other dataset. Thus, we project both ${\tilde{X}}^{R}$ and ${\tilde{X}}^{Q}$ into a low-dimensional space. Then, we look for inter-dataset graph connections with the MNN approach.

First, for each cell, we use the KNN method to identify its K nearest neighbors in the other dataset within the low-dimensional space. Then, for cell i in the reference data and cell j in the query data, if cell i is among the K nearest neighbors of cell j and also cell j is among the K nearest neighbors of cell i, $A_{ij}^{RQ}=1$. Otherwise $A_{ij}^{RQ}=0$. So, we get the inter-dataset graph connections $A^{RQ}$.

#### 2.2.4 Intra-dataset graph connections

The construction of intra-dataset graph connections is simpler. To accelerate the construction speed, we employ PCA for dimensionality reduction and, similar to the construction of inter-dataset graph connections, use MNN to construct intra-dataset connections. The intra-dataset connections for the reference data and the query data are denoted as $A^{RR}$ and $A^{QQ}$.

#### 2.2.5 Filtering of inter-dataset connections

We found that intra-dataset connections often have high accuracy. So, we consider using high-accuracy intra-dataset connections to filter inter-dataset connections.

Specifically, we first consider the intra-dataset connections of the reference data $A^{RR}$.We score each cell in $B^{R}$ through $A^{RR}$. For each cell r in the reference data, the score is given by


(2) $$u_{r}=\left\{ \begin{array}{c} \frac{\sum_{i\in N(r)}I_{\left[k_{R}(r)=k_{R}(i)\right]}}{\left|N(r)\right|}\;\;,\;\left|N(r)\right|\neq 0\\ 0\;\;\;\;\;\;\;\;\;\;\;\;\;\;\;\;\;\;\;\;,\;\left|N(r)\right|=0 \end{array}\right.$$

where N(r) is the set of adjacent cells of r in $A^{RR}$, $k_{R}(r)$is the cell type of r.

Based on these scores, we estimate the cell type of cell q which is in the cell set $B^{Q}$ using the connections $A^{RQ}$ between the reference data and the query data. The cell type with the highest score is selected as the estimated type for q.


(3) $$k_{Q}(q)=\left\{ \begin{array}{c} \underset{t\in T_{R}}{\arg\max}\left(\sum_{j\in N(q)}I_{\left[k_{R}(j)=t\right]}u_{j}\right),\;\text{if}\;\underset{t\in T_{R}}{\max}\left(\sum_{j\in N(q)}I_{\left[k_{R}(j)=t\right]}u_{j}\right)>0\\ “\text{Unknown}”\;\;\;\;\;\;\;\;\;\;\;\;,\;\text{other} \end{array}\right.$$

where $T_{R}$ is the set of cell types contained in the reference dataset, N(q) is the set of adjacent cells of q in $A^{RQ}$.

Then we use the information from the intra-dataset connections of the query data to re-estimate the type of q based on $A^{QQ}$.


(4) $$k_{Q}^{\prime }(q)=\underset{t\in T_{R}}{\arg\max}\sum_{j\in N(q)}I_{\left[k_{Q}(j)=t\right]}$$

where N(q) is the set of adjacent cells of q in $A^{QQ}$, which is usually the 4-hop neighborhoods of $A^{QQ}$ to expand the neighborhood range. To distinguish from the previous results, we use $k_{Q}^{\prime }(q)$ to record the newly estimated cell type of q.

Finally, for each connection in $A^{RQ}$ (where i denotes the cell in the reference data and j denotes the cell in the query data), if $k_{R}(i)\neq k_{Q}^{\prime }(j)$, the connection is considered unreliable, and remove the connection. So, we obtain the filtered inter-dataset connections $A^{RQ^{\prime }}$. Thus, the resulting hybrid graph is denoted as $A^{H}\in A^{RQ^{\prime }}\cup A^{RR}\cup A^{QQ}$.

#### 2.2.6 scGT algorithm

The scGT network is primarily composed of three modules: the encoder f(⋅), the GT module GT(⋅), and the classifier g(⋅).

$X^{R}$ and $X^{Q}$ first pass through an encoder, which consists of one fully connected layer mainly for dimensionality reduction.


(5) h=f(X)

where $X=\left[X^{R},X^{Q}\right]$.

For the GT module, we use the principle of Graph Transformer, which expands the receptive field to the entire graph, enabling information flow not only along edges but also globally. It mitigates the adverse effects of incorrect or incomplete connections. To accelerate training speed, we employ kernelized Gumbel-Softmax message passing (Wu *et al.* 2022). At layer l, the information passing is formulated as:


(6) $$q^{\left(l\right)}=W_{Q}^{\left(l\right)}h^{\left(l\right)},\;k^{\left(l\right)}=W_{K}^{\left(l\right)}h^{\left(l\right)},{\;v}^{\left(l\right)}=W_{V}^{\left(l\right)}h^{\left(l\right)}$$

(7) $$\begin{array}{c} h_{i}^{\left(l+1\right)}=\frac{\phi {\left(q_{i}^{\left(l\right)}/\sqrt{\tau }\right)}^{T}\sum_{j=1}^{\left(n_{R}+n_{Q}\right)}e^{g_{j}/\tau }\phi (k_{j}^{\left(l\right)}/\sqrt{\tau })\cdot {\left(v_{j}^{\left(l\right)}\right)}^{T}}{\phi {\left(q_{i}^{\left(l\right)}/\sqrt{\tau }\right)}^{T}\sum_{w=1}^{\left(n_{R}+n_{Q}\right)}e^{g_{w}/\tau }\phi (k_{w}^{\left(l\right)}/\sqrt{\tau })}\\ +\sum_{\left\{j|A_{i,j}^{H}=1\right\}}\sigma \left(\beta^{\left(l\right)}\right)\cdot v_{j} \end{array}$$

where similar to a transformer, $W_{Q}^{\left(l\right)}$, $W_{K}^{\left(l\right)}$, $W_{V}^{\left(l\right)}$ are the learnable weight matrices in layer l and $q^{\left(l\right)}$, $k^{\left(l\right)}$, $v^{\left(l\right)}$ are the obtained query matrix, key matrix, and value matrix. And $g_{j}$ follows a Gumbel distribution, τ is a temperature coefficient, σ(⋅) is an activation function, $\beta^{\left(l\right)}$ is a learnable parameter, ϕ(⋅) is a random feature map, and they use positive random feature (Krzysztof *et al.* 2021), i.e.


(8) $$\phi \left(x\right)=\frac{\;\text{exp}(\frac{-||x|{|}_{2}^{2}}{2})}{\sqrt{m}}\left[\text{exp}(w_{1}^{T}x),\ldots ,\;\text{exp}(w_{m}^{T}x)\right]$$

where $w_{k}∼N(0,I_{d})$ is i.i.d., sampled random transformation, m is a hyperparameter, which is fixed to 35 in our method.

Note that the first term in Equation (7) implements the global information flow, and the second term captures information from adjacent cells and implements the local information flow. For all the experiments presented in this article, we use two layers of kernelized Gumbel-Softmax message passing. Finally, the output of the GT module is


$$H=\left[H^{R},H^{Q}\right]=GT(h)\in R^{d\times (n_{R}+n_{Q})}.$$

Our integration goal is to minimize the differences between the reference data and the query data. To achieve this, we construct a hard regularization loss based on inter-dataset connections.


(9) $$L_{\mathrm{Hard}}=\max\left\{\left(\frac{1}{d\sum_{\left\{i,j|A_{i,j}^{RQ^{\prime }}=1\right\}}1}\sum_{\left\{i,j|A_{i,j}^{RQ^{\prime }}=1\right\}}{\left\|H_{i}^{R}-H_{j}^{Q}\right\|}_{2}^{2}\right)-\varepsilon ,\;0\right\}$$

where i and j are cells from the reference data and the query data, d is the dimension of H, and ε is a relaxation parameter, which is set to 0.1 by default.

Then, using a classifier to categorize the data. The classifier consists of a single fully connected layer designed to project the output embedding into a space with dimensionality equal to the number of categories in the reference data.


(10) C=g(H)

where $C\in R^{\left|T_{R}\right|\times (n_{R}+n_{Q})}$.

For each cell in $B^{R}$, we compute the cross-entropy loss using the output after the Softmax transformation to supervise the learning through the label of the reference data.


(11) $$P_{j,i}=\frac{\;\text{exp}(C_{j,i})}{\sum_{s=1}^{\left|T_{R}\right|}\;\text{exp}(C_{s,i})}$$

(12) $$L_{\mathrm{Entropy}}=-\frac{1}{\left|B^{R}\right|}\sum_{i\in B^{R}}\sum_{j=1}^{\left|T_{R}\right|}I_{\left[k_{R}(i)=j\right]}\;\text{log}\;P_{j,i}$$

where $C_{j,i}$ is the *j*th dimension output of cell i, $\left|T_{R}\right|$ is the number of categories in the reference data.

For cell q in query data, the predicted cell type is $k_{Q}^{\mathrm{pre}}(q)=\underset{t\in T_{R}}{\arg\max}P_{t,q}$, and the confidence score for the predicted type is $\mathrm{Confidence}(q)=\max\left\{P_{1,q},\ldots ,P_{|T_{R}|,q}\right\}$.

Additionally, to balance the impact of incorrect connections between datasets in the hard regularization loss, we construct query graph regularization.


(13) $$L_{\mathrm{Query}}=\max\left\{\left(1-\left(\frac{1}{\sum_{\left\{i,j|A_{i,j}^{QQ}=1\right\}}1}\sum_{\left\{i,j|A_{i,j}^{QQ}=1\right\}}I_{\left[k_{Q}^{\mathrm{pre}}(i)=k_{Q}^{\mathrm{pre}}(j)\right]}\right)\right)-\varepsilon ,\;0\right\}$$

where i and j are cells in the query data, and ε is a relaxation coefficient, which is set to 0.1 by default. This loss hopes that the predicted cell type at both ends of the intra-dataset graph connections in the query data to be the same. Considering that intra-dataset connections have a high accuracy, this helps to balance the adverse effects caused by incorrect inter-dataset connections and makes the model stop training at a more suitable time.

To sum up, the total loss is $L=L_{\mathrm{Hard}}+L_{\mathrm{Entropy}}+L_{\mathrm{Query}}$.

#### 2.2.7 (Optional) dynamic adjustment of supervisory signals

We observed that during the training process, the cells in the query dataset with confidence scores exceeding 0.9 are often correctly predicted. We consider using their predicted types as their true cell types and make these a new supervisory signal in the calculation of cross-entropy loss. To make this process more reliable, we set a high confidence score threshold of 0.95.

### 2.3 Evaluation metrics

#### 2.3.1 Silhouette coefficient

Silhouette coefficient (Rousseeuw 1987). First, we calculate the omics silhouette coefficient $Sil_{\mathrm{omic}}$ (based on omics) and cell type silhouette coefficient $Sil_{\mathrm{celltype}}$ (based on the ground-truth cell types). The larger the $Sil_{\mathrm{celltype}}$ is, the better, and the smaller the $Sil_{\mathrm{omic}}$ is, the better. For consistency, we usually use $1-Sil_{\mathrm{omic}}$. Then we calculate the F1 score of the silhouette coefficient as follows:


(14) $$Sil_{F1}=\frac{2\cdot \left[1-\left(1+Sil_{\mathrm{omic}}\right)/2\right]\cdot \left[\left(1+Sil_{\mathrm{celltype}}\right)/2\right]}{\left[1-\left(1+Sil_{\mathrm{omic}}\right)/2\right]+\left[\left(1+Sil_{\mathrm{celltype}}\right)/2\right]}$$

#### 2.3.2 Mean average precision

Mean average precision (MAP) (Cao and Gao 2022). It is used to evaluate the cell type resolution. We suppose that the *i*th cell’s cell type is $y^{\left(i\right)}$. And the cell types of the cell’s W ordered nearest neighbors are $y_{1}^{\left(i\right)},y_{2}^{\left(i\right)},\ldots ,y_{W}^{\left(i\right)}$. The average precision (AR) is defined as follows:


(15) $$AP^{\left(i\right)}=\left\{ \begin{array}{c} \frac{\sum_{s=1}^{W}I_{\left[y^{\left(i\right)}=y_{s}^{\left(i\right)}\right]}\cdot \frac{\sum_{t=1}^{s}I_{\left[y^{\left(i\right)}=y_{t}^{\left(i\right)}\right]}}{s}}{\sum_{s=1}^{W}I_{\left[y^{\left(i\right)}=y_{s}^{\left(i\right)}\right]}},\;\text{if}\;\sum_{s=1}^{W}I_{\left[y^{\left(i\right)}=y_{s}^{\left(i\right)}\right]}>0\\ 0\;\;\;\;\;\;\;\;\;\;\;\;\;\;\;\;\;\;\;\;\;\;\;\;\;\;\;,\;\;\text{other} \end{array}\right.$$

AP computes the average cell type precision up to each cell type-matched neighbor for each cell. Then for *N* cells, we defined MAP as follows:


(16) $$\mathrm{MAP}=\frac{1}{N}\sum_{i=1}^{N}AP^{\left(i\right)}$$

#### 2.3.3 Cell type average silhouette width

Cell type average silhouette width (cell type ASW) (Luecken *et al.* 2022). It is used to evaluate the cell type resolution. Defined as follows:


(17) $$\mathrm{cell}\;\mathrm{type}\;\mathrm{ASW}=\frac{1}{2}\cdot \left(\frac{\sum_{i=1}^{N}Sil_{\mathrm{celltype}}^{\left(i\right)}}{N}+1\right)$$

#### 2.3.4 Label transfer accuracy

We use label transfer accuracy to measure the accuracy of cell type prediction in query data (scATAC-seq). We defined it as follows:


(18) $$\mathrm{Accuracy}=\frac{\sum_{i=1}^{n_{Q}}\delta \left(k_{Q}(i),k_{Q}^{\mathrm{pre}}(i)\right)}{n_{Q}}$$

(19) $$\delta \left(k_{Q}(i),k_{Q}^{\mathrm{pre}}(i)\right)=\left\{ \begin{array}{c} 1,\;\;\;\;\text{if}\;k_{Q}(i)=k_{Q}^{\mathrm{pre}}(i)\\ 0,\;\;\;\;\text{other} \end{array}\right.$$

where $k_{Q}(i)$ is the true cell type of i, $k_{Q}^{\mathrm{pre}}(i)$ is the predicted cell type of cell i, and $n_{Q}$ is the cell counts of query data.

## 3 Results

### 3.1 scGT performs well on paired datasets

To evaluate the integration and label transfer ability of scGT on single-cell multi-omics datasets, we first applied it to two datasets obtained from different paired sequencing technologies, including the SNARE-seq data of the mouse brain cortex (Chen *et al.* 2019) and the SHARE-seq data of the mouse colon (Ma *et al.* 2020b). The two datasets are from different tissues. We treated the paired data as two separate omics and did not use pairwise information.

**Figure 2.**
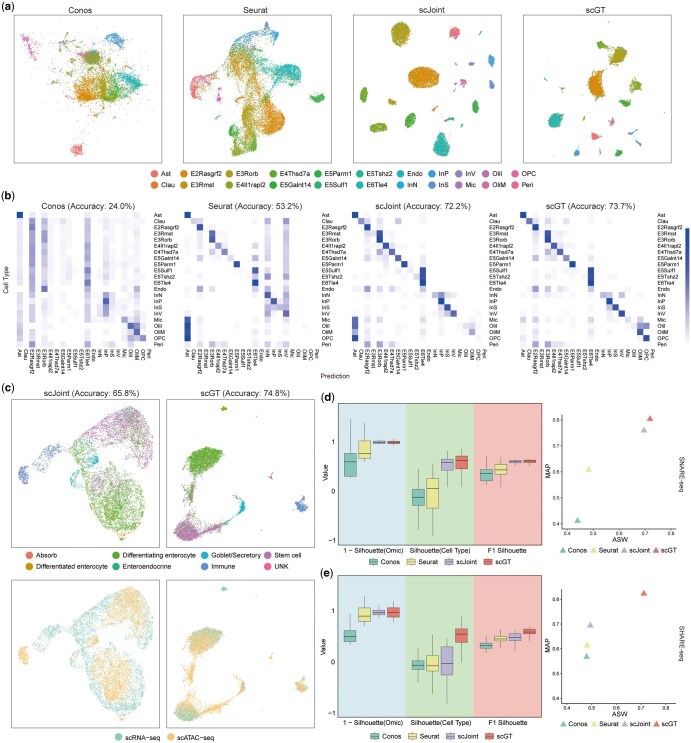
scGT performs well on paired datasets. (a) UMAP visualization of four methods on SNARE-seq mouse brain cortex data, colored by cell types. (b) Heatmap of normalized label transfer confusion matrix for four methods on SNARE-seq mouse brain cortex data. (c) UMAP visualization of scJoint and scGT on SHARE-seq mouse colon data, colored by cell types and omics. (d) The cell type, 1-omics silhouette coefficients, and F1 score of silhouette coefficients (left) and ASW-MAP scatter plot (right) for scGT and other baselines on SNARE-seq mouse brain cortex data. Each boxplot ranges from the upper and lower quartiles, with the median as the horizontal line. (e) The cell type, 1-omics silhouette coefficients, and F1 score of silhouette coefficients (left) and ASW-MAP scatter plot (right) for scGT and other baselines on SHARE-seq mouse colon data. Each boxplot ranges from the upper and lower quartiles, with the median as the horizontal line.

The scGT integrated the two paired datasets well, enabling better aggregation of cells with the same type and achieving the highest label transfer accuracy. On the mouse brain cortex dataset, scGT’s label transfer accuracy is 73.7%, which is 1.5% higher than scJoint, 20.5% higher than Seurat, and 49.7% higher than Conos. Compared with other methods, scGT predicted OliM and OPC cells more accurately, and the heatmap of the normalized confusion matrix had a clearer diagonal structure (Fig. 2b). On the mouse colon dataset, the label transfer accuracy of scGT is 9.0% higher compared to scJoint (Fig. 2c). scGT downscaled the learned joint embedding to get the joint visualization, and the UMAP visualization showed that scGT fused the data from different omics well and better separated different cell types (Fig. 2a and c and Fig. 1, available as supplementary data at *Bioinformatics* online). The quantitative evaluation metrics confirmed this observation. On both datasets, scGT achieved the best cell type silhouette coefficients and F1 scores of silhouette coefficients. Omics silhouette coefficients were similar to scJoint. To verify the integration effect more rigorously, we further calculated MAP and cell type ASW, and scGT achieved the highest scores (Fig. 2d and e).

In conclusion, the results on the paired datasets show that scGT integrates data from different omics well and achieves high accuracy in label transfer.

### 3.2 scGT performs well on unpaired and type-matched datasets

The integration and label transfer ability of scGT on paired datasets were assessed in the above experiments. Here, we further explored the performance of scGT on the type-matched unpaired single-cell multi-omics dataset. We evaluated it on the PBMC dataset (Mimitou *et al.* 2021) and only used the gene expression matrix and gene activity matrix. It has seven cell types and contains 4644 scRNA-seq data and 4157 scATAC-seq data.

**Figure 3.**
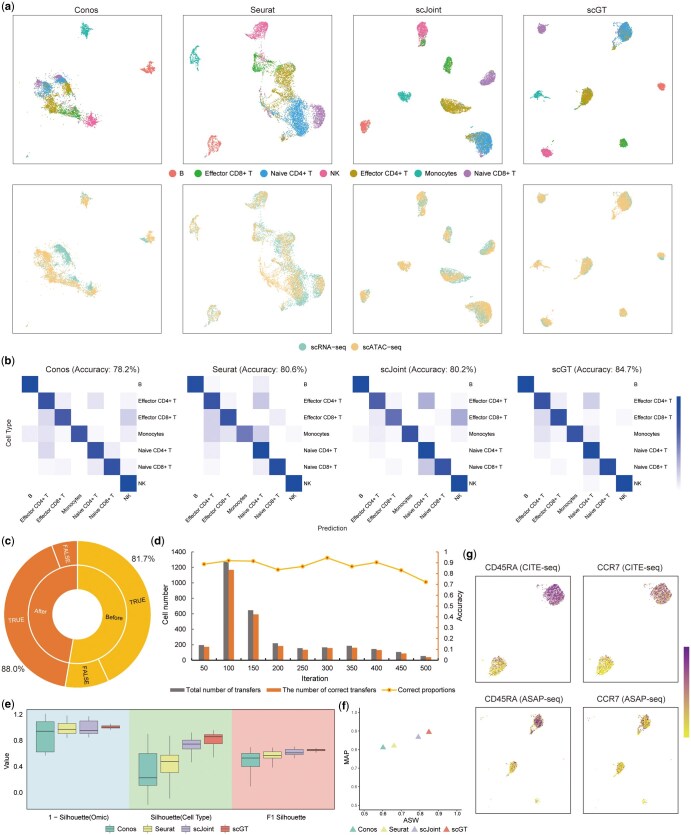
scGT performs well on unpaired and type-matched datasets. (a) UMAP visualization of four methods colored by cell types and omics. (b) Heatmap of normalized label transfer confusion matrix for four methods on PBMC data. (c) Comparison of correct-error cases before and after filtering inter-dataset connections using scGT. (d) Plot of accuracy for transfer cells (prediction confidence score > 0.95) in the query dataset during the scGT training period. (e) The cell type, 1-omics silhouette coefficients, and F1 score of silhouette coefficients for scGT and three baselines on PBMC data. Each boxplot ranges from the upper and lower quartiles, with the median as the horizontal line. (f) ASW-MAP scatter plot on PBMC data. (g) Expression of CCR7 gene and CD45RA protein on Naive CD4+ T cells and Effector CD4+ T cells from CITE-seq and ASAP-seq data.

Among all the methods, scGT had the highest label transfer accuracy (84.7%), while scJoint was 80.2%, Seurat was 80.6%, and Conos was 78.2% (Fig. 3b). Compared to Conos and Seurat, scGT and scJoint exhibit more distinct cell clusters (Fig. 3a). Meanwhile, scGT had higher silhouette coefficients, ASW, and MAP compared to scJoint (Fig. 3e and f).

We evaluated the effectiveness of scGT’s inter-dataset connection filtering method by defining a connection as correct if the cell types at both endpoints were consistent and incorrect otherwise. In this dataset, the initial inter-dataset connections constructed from the original data included 4469 correct connections and 1001 incorrect connections, yielding a correctness rate of 81.7%. After applying the filtering step using high-accuracy intra-dataset connections, the number of correct connections became 4321, while incorrect connections decreased to 590, increasing to a correctness rate of 88.0% (Fig. 3c). It indicates that our filtering method for inter-dataset connections in the hybrid graph effectively drops most incorrect connections while preserving the correct connections as much as possible. This approach enhances the quality of the hybrid graph, improving the integration effectiveness of subsequent algorithms and the accuracy of label transfer. To further evaluate the robustness of the strategy of including cells with predicted confidence scores exceeding 0.95 in the subsequent cross-entropy loss computation, we tracked the number of correct and incorrect predictions for the added cells in each operation during the scGT training period (Fig. 3d). During the training process, a significant proportion of the moved cells was correctly predicted, and the process remained stable throughout.

Finally, we show the expression of the CCR7 gene and the CD45RA protein in Naive CD4+ T cells and Effector CD4+ T cells in CITE-seq and ASAP-seq (Fig. 3g). CD45RA and CCR7 are typically highly expressed in Naive CD4+ T cells and exhibit low expression in Effector CD4+ T cells (Sallusto *et al.* 1999, 2004). The gene and protein expression in different omics were consistent with this conclusion, supporting the plausibility of scGT’s clusters and the accuracy of its predictions.

### 3.3 scGT effectively integrates datasets with mismatched types

The above experiments’ cell types in both scRNA-seq and scATAC-seq data are identical. However, in practical applications, multi-omics datasets often contain mismatched cell types. Therefore, it is necessary to explore the capability of scGT in integrating single-cell multi-omics data with mismatched cell types and in transferring labels based on these datasets. We explored it on human myocardial infarction data (Kuppe *et al.* 2022). The snRNA-seq data have 42 458 cells and 10 cell types, and the snATAC-seq data have 46 086 cells and 8 cell types. Specifically, only seven cell types are common to the snRNA-seq and snATAC-seq data. Mast, Adipocyte, and Cycling cells are only present in the snRNA-seq data, and Myeloid cells are only in the snATAC-seq. They all have unique cell types.

**Figure 4.**
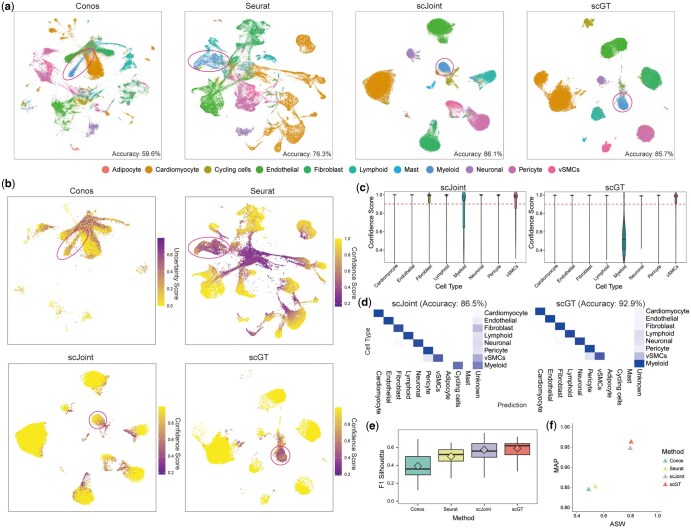
scGT effectively integrates datasets with mismatched types. (a) UMAP visualization of four methods colored by cell types. Red circles show the location of Myeloid cells. (b) The confidence scores (uncertainty scores) for four methods in the UMAP visualization of snATAC-seq data. Red circles show the location of Myeloid cells. (c) Violin plots of confidence scores for scJoint and scGT categorized by cell types. (d) Heatmap of corrected and normalized label transfer confusion matrix for scJoint and scGT, taking a threshold of 0.9. Cells with a confidence score <0.9 are classified as Unknown type. (e) F1 score of silhouette coefficients for scGT and three baselines. Each boxplot ranges from the upper and lower quartiles, with the median as the horizontal line. The rhombus represents the average value. (f) ASW-MAP scatter plot.

In this case, we noted that separate clusters of Myeloid cells are observed in the UMAP visualizations of all four methods (Fig. 4a). However, scGT and scJoint were able to better separate the Myeloid cluster from other cells and kept Mast, Adipocyte, and Cycling cells as much as possible from mixing with snATAC-seq data, which Conos and Seurat did not achieve (Fig. 4a). Meanwhile, we noted that Seurat, scJoint, and scGT all get prediction confidence scores for cells, and Conos gets uncertainty scores. We analyzed them and observed that the cells with low confidence scores obtained by scGT were basically concentrated in the location where Myeloid cells were located, while Conos, Seurat, and scJoint were scattered everywhere (Fig. 4b). Seurat did not predict cell types contained in both omics with high confidence scores. scJoint still made some predictions with a confidence score >0.9 for the ‘Unknown’ cell type (Myeloid). It suggested that scJoint does not recognize unknown cell types well and that there are many false predictions with high confidence scores for unknown cell types. However, scGT accurately predicts cells with shared types between snRNA-seq and snATAC-seq data with high confidence scores while assigning low confidence scores to “Unknown” cells (Fig. 4c). It minimizes unwanted misclassification. We set a threshold of 0.9 for classification, designating cells below this value as “Unknown.” Notably, scGT effectively classified Myeloid cells as ‘Unknown’, while scJoint only partially differentiated them. Based on this, the corrected label transfer accuracy was 92.9% for scGT and 86.5% for scJoint (Fig. 4d). And we calculated the F1 score of silhouette coefficients, as well as the ASW and MAP, for all four methods, and scGT achieved the best results (Fig. 4e and f).

Further, we additionally removed Neuronal cells from the snRNA-seq data so that Neuronal and Myeloid cells were only in the snATAC-seq data. The results showed that scJoint failed to continue to separate Myeloid cells into a separate cluster after additional removal of Neuronal cells. In contrast, in scGT’s joint visualization, Myeloid cells were still able to be divided into a cluster independently (Fig. 2a, available as supplementary data at *Bioinformatics* online). Both scJoint and scGT were unable to separate Neuronal cells into a separate cluster because Neuronal cells have a small number of cells, but most of the Neuronal cells were in the region with low confidence scores in scGT (Fig. 2b, available as supplementary data at *Bioinformatics* online). scGT still accurately predicted cells with shared types between snRNA-seq and snATAC-seq data with high confidence scores and assigned low confidence scores to “Unknown” cells (Fig. 2d, available as supplementary data at *Bioinformatics* online). And we got a similar result by going one step further and removing the Cardiomyocyte cells (Fig. 3, available as supplementary data at *Bioinformatics* online).

Overall, scGT is excellent at handling type-mismatched datasets, identifying unknown cell types, avoiding misclassification as much as possible, and performs better overall in metrics.

### 3.4 scGT integrates complex atlas-scale scRNA-seq and scATAC-seq data

**Figure 5.**
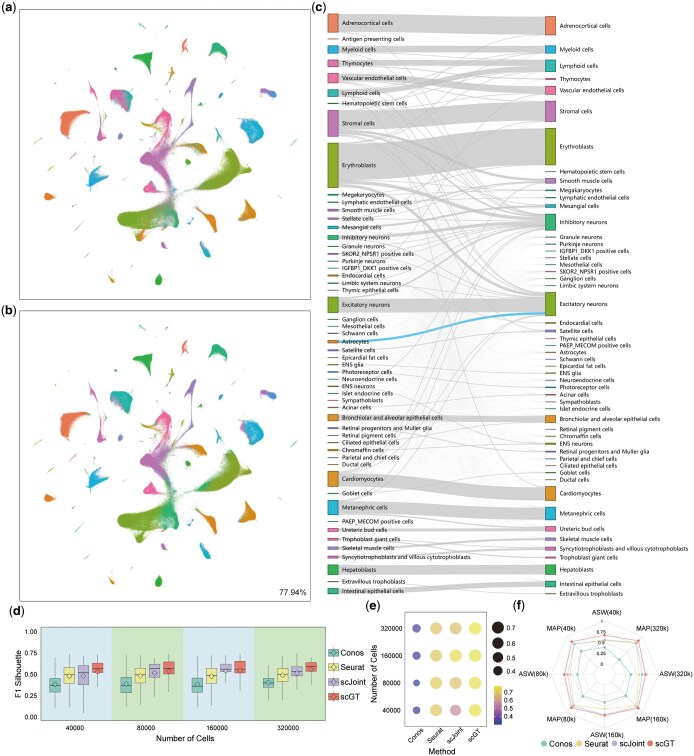
scGT integrates complex atlas-scale scRNA-seq and scATAC-seq data. (a) UMAP visualization of scGT on human fetal atlas data, colored by cell types (the color of cell types is consistent with c). (b) UMAP visualization of scGT on human fetal atlas data, colored by predicted types (the color of cell types is consistent with c). (c) Sankey diagram of scATAC-seq data, with cell types (left) and scGT predicted types (right). (d) F1 score of silhouette coefficients for scGT and three baselines on the 40 000, 80 000, 160 000, and 320 000 sampling datasets. Each boxplot ranges from the upper and lower quartiles, with the median as the horizontal line. The rhombus represents the average value. (e) Bubble Chart of label transfer accuracy for the four methods on the 40 000, 80 000, 160 000, and 320 000 sampling datasets. (f) ASW and MAP for the four methods on the 40 000, 80 000, 160 000, and 320 000 sampling datasets.

With the rapid advancement of single-cell sequencing technology, many large-scale atlas datasets have emerged. To evaluate the capability of scGT in handling large-scale atlas datasets, we selected human fetal atlas data (Cao *et al.* 2020, Domcke *et al.* 2020). We selected 54 cell types common to both scRNA-seq and scATAC-seq human fetal atlas datasets, and performed random sampling for those cell types with more than 10 000 cells in the scRNA-seq data. Finally, we got 433 695 scRNA-seq cells and 656 074 scATAC-seq cells, for a total of 1 089 769 cells.scGT successfully integrated millions of data, separated different cell types, and achieved effective label transfer with an accuracy of 77.94% (Fig. 5a and b). We note that scGT predicted the majority of Astrocytes (6775 cells) as Excitatory neurons, with 5560 having a confidence score >0.8 (Fig. 5c). We visualized cells initially labeled as Astrocytes and Excitatory neurons in the scRNA-seq and scATAC-seq data based on the joint embedding obtained by scGT. In the joint visualization, a small proportion of Excitatory neurons in scRNA-seq data were mixed with the majority of Astrocytes in scATAC-seq data. They were separated from the cluster composed of other Excitatory neurons (Fig. 5a and b, available as supplementary data at *Bioinformatics* online). Further examining gene expression, we found that the classical progenitor cell markers SOX2, PAX6, and HES1 were actively expressed in this cluster, while EOMES was expressed in small amounts (Pollen *et al.* 2015, Thomsen *et al.* 2016). The cluster also actively transcribed TNC, FAM107A, HOPX, and LIFR, which are gene markers for oRGs, and EGFR, which is a gene marker for tRGs (Yang *et al.* 2022b). It barely expressed ANXA1, a gene marker for vRGs (Thomsen *et al.* 2016). Therefore, we speculate that the cluster may not be Astrocytes or Excitatory neurons but may be multipotent neural progenitors, and the majority of them are likely to be oRGs (Outer radial glial cells) and tRGs (Truncated radial glial cells, Figs 5 and 6, available as supplementary data at *Bioinformatics* online).

Meanwhile, to explore the ability of scGT to integrate complex atlas data even with a small dataset, we randomly sampled 40 000, 80 000, 160 000, and 320 000 cells (the total number of scRNA-seq and scATAC-seq data) from human fetal atlas data. scGT achieved the highest label transfer accuracy across all cases (Fig. 5e), along with the highest ASW, MAP, and F1 score of silhouette coefficient (Fig. 5d and f). It demonstrates that scGT performs well in label transfer in complex atlas data while performing better fusion of omics and separation of different cell types, striking a balance between removing omics differences and preserving biosignatures. Even with only 40 000 cells used for integration, with 16 000 for scRNA-seq and 24 000 for scATAC-seq, scGT still integrated well and achieved the highest label transfer accuracy of 76.51% compared to 56.48% for scJoint (Fig. 4, available as supplementary data at *Bioinformatics* online).

This proves that scGT is capable of integrating complex atlas data with millions of cells. Even when the amount of data used for integration is small, the integration is still efficient and accurate.

## 4 Discussion

Currently, more than 1.5 million cells are sequenced and archived each month through various cell atlas projects (Svensson *et al.* 2020). As sequencing costs continue to decline, the volume of sequenced cells is growing rapidly (Angerer *et al.* 2017). However, the process of cell annotation still relies heavily on manual efforts, which hampers reproducibility and introduces biases into the data. While several open-access solutions have been developed to facilitate this process, they vary in accuracy (Argelaguet *et al.* 2021, Lee *et al.* 2023). Notably, deep-learning techniques that utilize transformer-based architectures for analyzing gene expression data have demonstrated superior performance compared to traditional methods (Yang *et al.* 2022a). However, these approaches require advanced computational knowledge and complicated deep neural networks. scGT, on the other hand, is trained efficiently with a semi-supervised training regime, avoiding complex computational knowledge. Furthermore, compared to the versatile neural network-based method scJoint, which aligns latent spaces to integrate multi-omics datasets, scGT is based on a graph transformer with the help of a hybrid graph that explicitly models both intra-dataset and inter-dataset relationships to enable effective multi-omics integration. With its global and local modeling capability, scGT improves cell type recognition and generalizes well to unseen cell types, making it a powerful tool for single-cell analysis.

From a technical perspective, scGT has some major advantages: scGT is a flexible model for diagonal integration of single-cell multi-omics data, in contrast to many established models for multi-omics data integration, our method initially utilize original data to construct a hybrid graph containing intra-dataset and inter-dataset connections. In practice, we found that the intra-dataset connections tend to have a high accuracy rate, so we filter the inter-dataset connections using the high-accuracy intra-dataset connections, aiming to retain the correct connections and remove as many erroneous connections as possible, ultimately obtaining a robust hybrid graph. Subsequently, scGT adopts the self-attention mechanism to extend the receptive field of node information flow from local neighborhoods to the entire graph, making the overall learning process both based on the graph structure and not entirely based on the graph structure, realizing information flow both along the edges and across the whole graph. While benefiting from the effective prior provided by the hybrid graph, the impact of erroneous connections in the hybrid graph is reduced as much as possible to enhance the algorithm’s performance. scGT defines three loss terms, cross-entropy loss, hard regularization loss, and query graph regularization, to co-train labeled scRNA-seq data and unlabeled scATAC-seq data, and achieve remarkably well multi-omics integration and label transfer. scGT obtains higher-quality joint embedding and superior label transfer accuracy in five experiments with multi-omics data than other methods. scGT also performs well in type-mismatched datasets, successfully identifying unknown cells and isolating unique cell clusters. scGT is able to handle complex atlas-scale data, and even at very small data sizes, scGT still shows good performance.

Although scGT requires constructing and filtering hybrid graphs, we simplify its implementation by packaging the hybrid graph construction and graph connection filtering parts of scGT. We simply import the gene expression matrix, gene activity matrix, and cell types, after that, we obtain the inputs for the subsequent network through the function conveniently. Our filtering method has shown promising results, effectively addressing over-filtering for most cell types. However, we recognize that cell types with small cell counts may still be slightly affected, and we aim to refine this aspect in future work to optimize the method further.

In summary, scGT is a multi-omics data integration method based on a novel paradigm, not only for omics alignment but also label transfer, and scGT shows an impressive performance in identifying cell types and preserving biological variation. Our strategy of graph construction aids in enhancing the refinement of the cell graph and inferring cell representations. We believe that scGT, being a modular framework, offers a unique opportunity to effectively outline unmatched and diverse cell types through large-scale multi-omics integration at the single cell.

## Supplementary Material

btaf357_Supplementary_Data

## References

[btaf357-B1] Angerer P , Simon L, Tritschler S et al Single cells make big data: new challenges and opportunities in transcriptomics. Curr Opin Syst Biol 2017;4:85–91.

[btaf357-B2] Argelaguet R , Cuomo ASE, Stegle O et al Computational principles and challenges in single-cell data integration. Nat Biotechnol 2021;39:1202–15. 33941931 10.1038/s41587-021-00895-7

[btaf357-B3] Argelaguet R , Arnol D, Bredikhin D et al MOFA+: a statistical framework for comprehensive integration of multi-modal single-cell data. Genome Biol 2020;21:111. 32393329 10.1186/s13059-020-02015-1 PMC7212577

[btaf357-B4] Barkas N , Petukhov V, Nikolaeva D et al Joint analysis of heterogeneous single-cell RNA-seq dataset collections. Nat Methods 2019;16:695–8. 31308548 10.1038/s41592-019-0466-z PMC6684315

[btaf357-B5] Berger SL. The complex language of chromatin regulation during transcription. Nature 2007;447:407–12. 17522673 10.1038/nature05915

[btaf357-B6] Cao J , O’Day DR, Pliner HA et al A human cell atlas of fetal gene expression. Science 2020;370:eaba7721. 33184181 10.1126/science.aba7721 PMC7780123

[btaf357-B7] Cao J , Cusanovich DA, Ramani V et al Joint profiling of chromatin accessibility and gene expression in thousands of single cells. Science 2018;361:1380–5. 30166440 10.1126/science.aau0730 PMC6571013

[btaf357-B8] Cao Z-J , Gao G. Multi-omics single-cell data integration and regulatory inference with graph-linked embedding. Nat. Biotechnol 2022;40:1458–66. 35501393 10.1038/s41587-022-01284-4 PMC9546775

[btaf357-B9] Chen S , Lake BB, Zhang K et al High-throughput sequencing of the transcriptome and chromatin accessibility in the same cell. Nat Biotechnol 2019;37:1452–7. 31611697 10.1038/s41587-019-0290-0 PMC6893138

[btaf357-B10] Dimitrakopoulos C , Hindupur SK, Häfliger L et al Network-based integration of multi-omics data for prioritizing cancer genes. Bioinformatics 2018;34:2441–8. 29547932 10.1093/bioinformatics/bty148 PMC6041755

[btaf357-B11] Domcke S , Hill AJ, Daza RM et al A human cell atlas of fetal chromatin accessibility. Science 2020;370:eaba7612. 33184180 10.1126/science.aba7612 PMC7785298

[btaf357-B12] Haghverdi L , Lun ATL, Morgan MD et al Batch effects in single-cell RNA-sequencing data are corrected by matching mutual nearest neighbors. Nat Biotechnol 2018;36:421–7. 29608177 10.1038/nbt.4091 PMC6152897

[btaf357-B13] Hotelling H. Analysis of a complex of statistical variables into principal components. J. Educ. Psychol 1933;24:417–41.

[btaf357-B14] Jin S , Zhang L, Nie Q et al scAI: an unsupervised approach for the integrative analysis of parallel single-cell transcriptomic and epigenomic profiles. Genome Biol 2020;21:25. 32014031 10.1186/s13059-020-1932-8 PMC6996200

[btaf357-B15] Kalisky T , Oriel S, Bar-Lev TH et al A brief review of single-cell transcriptomic technologies. Brief Funct Genomics 2018;17:64–76. 28968725 10.1093/bfgp/elx019

[btaf357-B16] Klemm SL , Shipony Z, Greenleaf WJ et al Chromatin accessibility and the regulatory epigenome. Nat Rev Genet 2019;20:207–20. 30675018 10.1038/s41576-018-0089-8

[btaf357-B17] Krzysztof C, Valerii L, David D et al Rethinking attention with performers. In: *9th International Conference on Learning Representations*. OpenReview.net, 2021.

[btaf357-B18] Kuppe C , Ramirez Flores RO, Li Z et al Spatial multi-omic map of human myocardial infarction. Nature 2022;608:766–77. 35948637 10.1038/s41586-022-05060-x PMC9364862

[btaf357-B19] Lee MYY , Kaestner KH, Li M et al Benchmarking algorithms for joint integration of unpaired and paired single-cell RNA-seq and ATAC-seq data. Genome Biol 2023;24:244. 37875977 10.1186/s13059-023-03073-x PMC10594700

[btaf357-B20] Li Y , Zhang D, Yang M et al scBridge embraces cell heterogeneity in single-cell RNA-seq and ATAC-seq data integration. Nat Commun 2023;14:6045. 37770437 10.1038/s41467-023-41795-5 PMC10539354

[btaf357-B21] Lin Y , Wu T-Y, Wan S et al scJoint integrates atlas-scale single-cell RNA-seq and ATAC-seq data with transfer learning. Nat Biotechnol 2022;40:703–10. 35058621 10.1038/s41587-021-01161-6 PMC9186323

[btaf357-B22] Luecken MD , Büttner M, Chaichoompu K et al Benchmarking atlas-level data integration in single-cell genomics. Nat Methods 2022;19:41–50. 34949812 10.1038/s41592-021-01336-8 PMC8748196

[btaf357-B23] Ma A , McDermaid A, Xu J et al Integrative methods and practical challenges for Single-Cell multi-omics. Trends Biotechnol 2020a;38:1007–22. 32818441 10.1016/j.tibtech.2020.02.013 PMC7442857

[btaf357-B24] Ma S , Zhang B, LaFave LM et al Chromatin potential identified by shared Single-Cell profiling of RNA and chromatin. Cell 2020b;183:1103–16.e20. 33098772 10.1016/j.cell.2020.09.056 PMC7669735

[btaf357-B25] Mimitou EP , Lareau CA, Chen KY et al Scalable, multimodal profiling of chromatin accessibility, gene expression and protein levels in single cells. Nat Biotechnol 2021;39:1246–58. 34083792 10.1038/s41587-021-00927-2 PMC8763625

[btaf357-B26] Picelli S , Björklund ÅK, Faridani OR et al Smart-seq2 for sensitive full-length transcriptome profiling in single cells. Nat Methods 2013;10:1096–8. 24056875 10.1038/nmeth.2639

[btaf357-B27] Pollen AA , Nowakowski TJ, Chen J et al Molecular identity of human outer radial glia during cortical development. Cell 2015;163:55–67. 26406371 10.1016/j.cell.2015.09.004 PMC4583716

[btaf357-B28] Pott S , Lieb JD. Single-cell ATAC-seq: strength in numbers. Genome Biol 2015;16:172. 26294014 10.1186/s13059-015-0737-7 PMC4546161

[btaf357-B29] Rousseeuw PJ. Silhouettes: a graphical aid to the interpretation and validation of cluster analysis. J Comput Appl Math 1987;20:53–65.

[btaf357-B30] Sallusto F , Geginat J, Lanzavecchia A et al Central memory and effector memory T cell subsets: function, generation, and maintenance. Annu Rev Immunol 2004;22:745–63. 15032595 10.1146/annurev.immunol.22.012703.104702

[btaf357-B31] Sallusto F , Lenig D, Förster R et al Two subsets of memory T lymphocytes with distinct homing potentials and effector functions. Nature 1999;401:708–12. 10537110 10.1038/44385

[btaf357-B32] Song Q , Su J, Zhang W et al scGCN is a graph convolutional networks algorithm for knowledge transfer in single cell omics. Nat Commun 2021;12:3826. 34158507 10.1038/s41467-021-24172-y PMC8219725

[btaf357-B33] Stuart T , Butler A, Hoffman P et al Comprehensive integration of single-cell data. Cell 2019;177:1888–902.e21. 31178118 10.1016/j.cell.2019.05.031 PMC6687398

[btaf357-B34] Svensson V , da Veiga Beltrame E, Pachter L et al A curated database reveals trends in single-cell transcriptomics. Database J Biol Databases Curation 2020;2020:baaa073. 10.1093/database/baaa073 PMC7698659 33247933

[btaf357-B35] Tabula Muris Consortium et al Single-cell transcriptomics of 20 mouse organs creates a tabula muris. Nature 2018;562:367–72. 30283141 10.1038/s41586-018-0590-4 PMC6642641

[btaf357-B36] Thomsen ER , Mich JK, Yao Z et al Fixed single-cell transcriptomic characterization of human radial glial diversity. Nat Methods 2016;13:87–93. 26524239 10.1038/nmeth.3629 PMC4869711

[btaf357-B37] Wu Q, Zhao W, Li Z et al NodeFormer: a scalable graph structure learning transformer for node classification. In: Koyejo S et al (eds), Advances in Neural Information Processing Systems. Red Hook, New York: Curran Associates, Inc., 2022, 27387–401.

[btaf357-B38] Yang F , Wang W, Wang F et al scBERT as a large-scale pretrained deep language model for cell type annotation of single-cell RNA-seq data. Nat Mach Intell 2022a;4:852–66.

[btaf357-B39] Yang L , Li Z, Liu G et al Developmental origins of human cortical oligodendrocytes and astrocytes. Neurosci Bull 2022b;38:47–68. 34374948 10.1007/s12264-021-00759-9 PMC8783027

[btaf357-B40] Zhang X, Zhang S, Zhang X et al Fast virtual stenting for thoracic endovascular aortic repair of aortic dissection using graph deep learning. IEEE J Biomed Health Inform 2025;29:4374–87. 40036417 10.1109/JBHI.2025.3540712

[btaf357-B41] Zheng GXY , Terry JM, Belgrader P et al Massively parallel digital transcriptional profiling of single cells. Nat Commun 2017;8:14049. 28091601 10.1038/ncomms14049 PMC5241818

