# Supplementary material for: scGT:integration algorithm for single-cell RNA-seq and ATAC-seq based on graph transformer
Source: Bioinformatics. 2025 Jun 24;41(7):btaf357. doi: 10.1093/bioinformatics/btaf357 (PMC12233090; doi:10.1093/bioinformatics/btaf357)
Supplement: btaf357_Supplementary_Data [file btaf357_supplementary_data.pdf]

SUPPLEMENTARY INFORMATION

scGT: Integration algorithm for single-cell RNA-seq and ATAC-seq

based on Graph Transformer

Supplementary Figures ------------1-6
Supplementary Notes ------------7-9
Reference -------------10

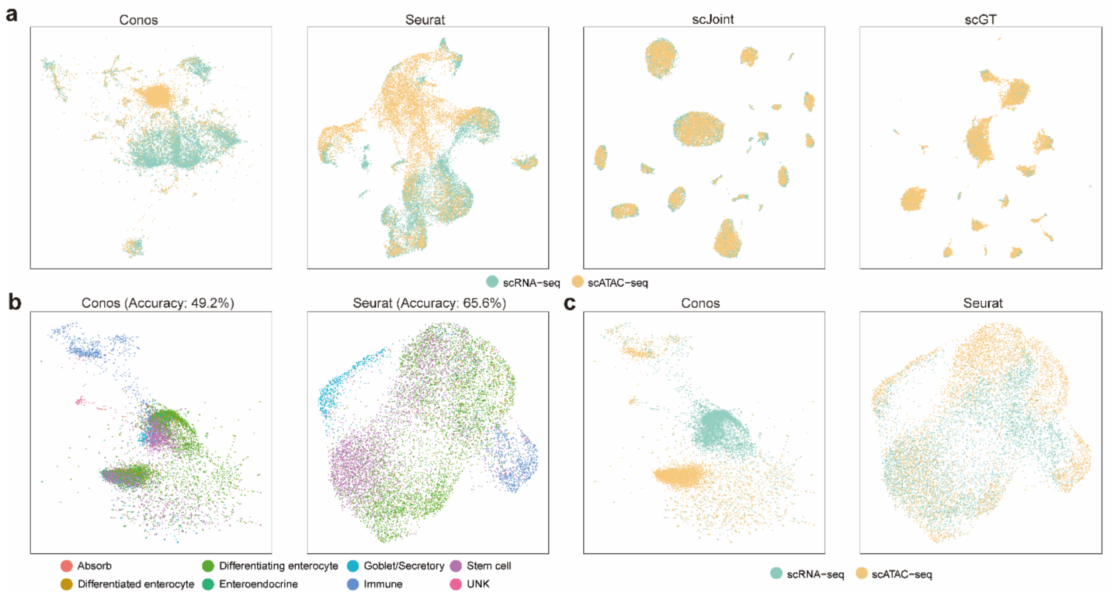

Supplementary Fig. 1 Results of paired datasets. a UMAP visualization of four methods on SNARE-seq mouse brain cortex data, colored by omics. **b** UMAP visualization of Conos and Seurat on SHARE-seq mouse colon data, colored by cell types. **c** UMAP visualization of Conos and Seurat on SHARE-seq mouse colon data, colored by omics.

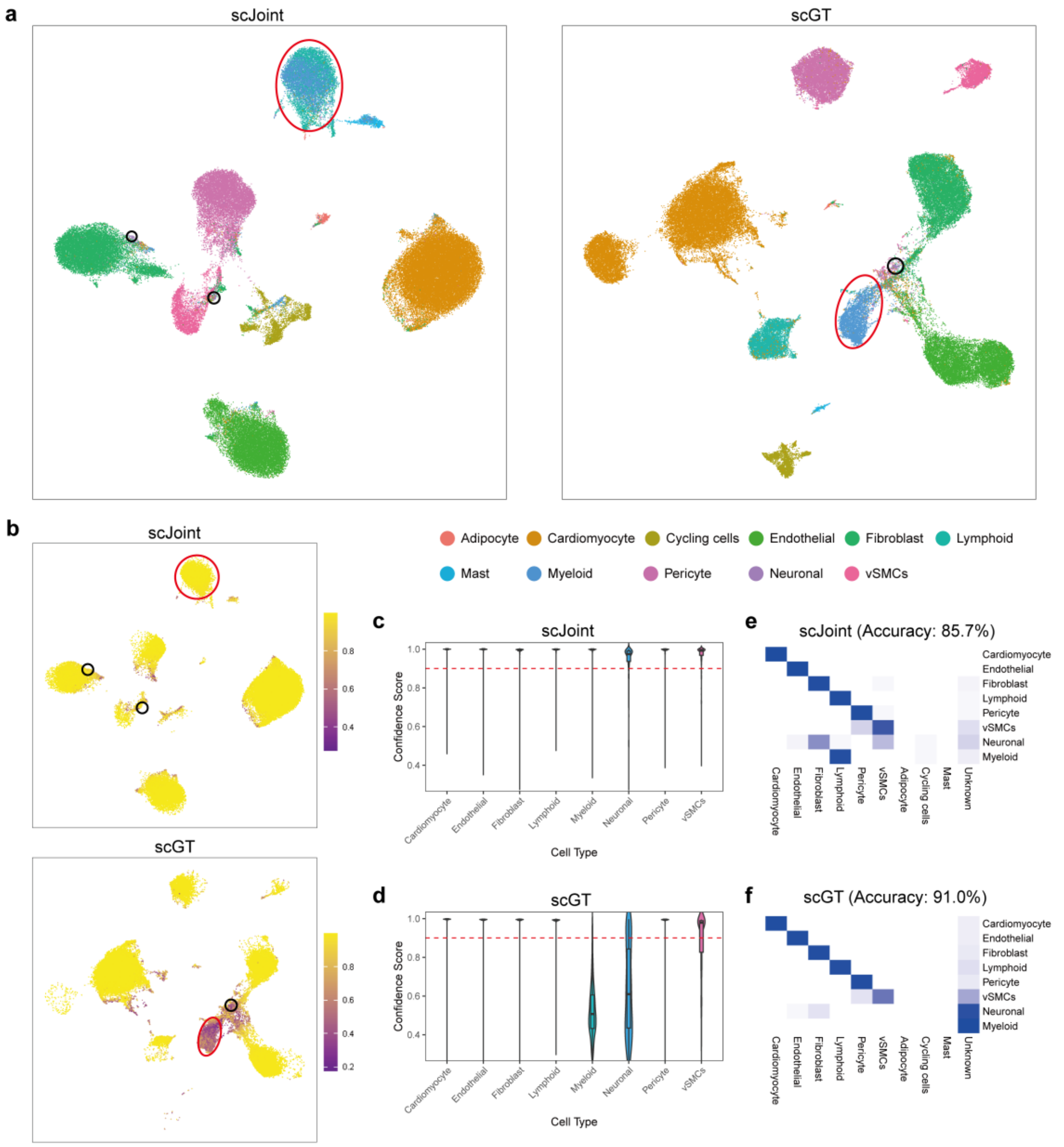

Supplementary Fig. 2 Neuronal and Myeloid cells are only in snATAC-seq on human myocardial infarction data. **a** UMAP visualization of scJoint and scGT colored by cell types. Red circles show the location of Myeloid cells and black circles show the location of Neuronal cells. **b** The confidence scores for scJoint and scGT in the UMAP visualization of snATAC-seq data. Red circles show the location of Myeloid cells and black circles show the location of Neuronal cells. **c** Violin plots of confidence scores for scJoint categorized by cell types. **d** Violin plots of confidence scores for scGT categorized by cell types. **e** Heatmap of corrected and normalized label transfer confusion matrix for scJoint, taking a threshold of 0.9. Cells with a confidence score less than 0.9 are classified as Unknown type. **f** Heatmap of corrected and normalized label transfer confusion matrix for scGT, taking a threshold of 0.9. Cells with a confidence score less than 0.9 are classified as Unknown type.

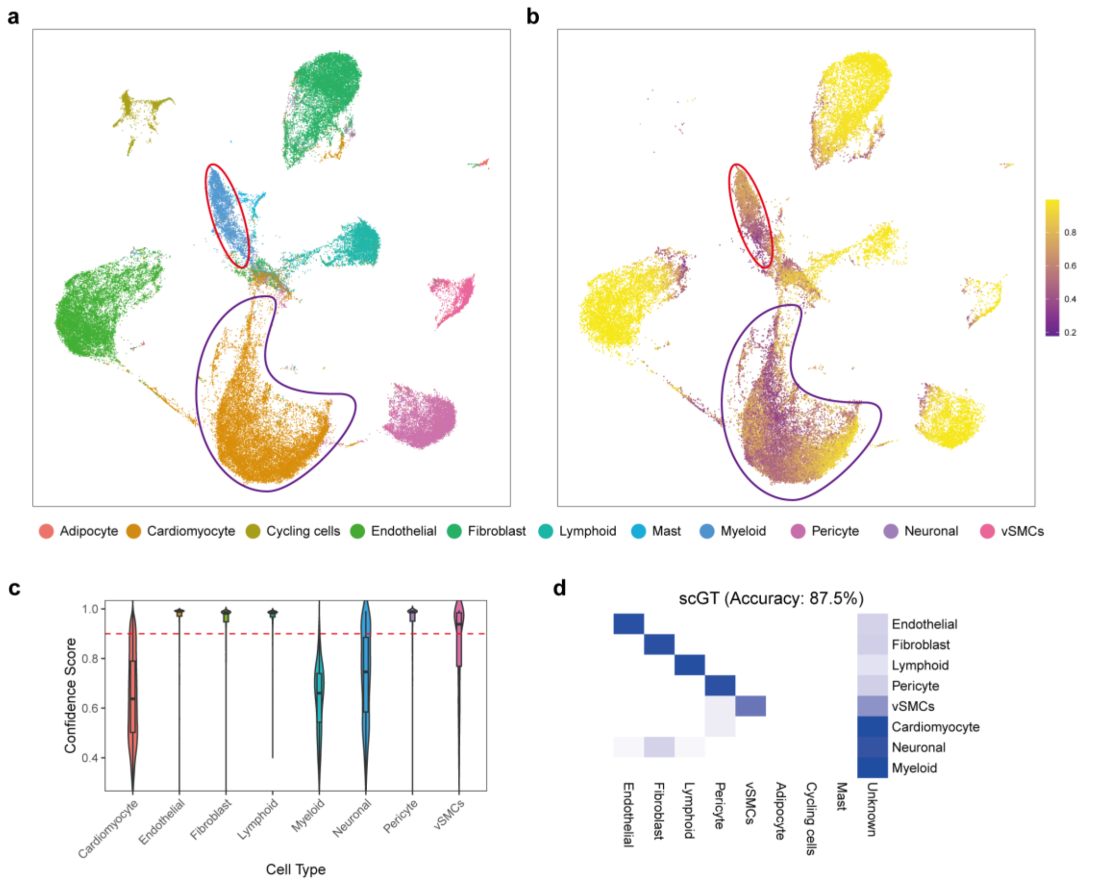

Supplementary Fig. 3 Cardiomyocyte, Neuronal and Myeloid cells are only in snATAC-seq on human myocardial infarction data. **a** UMAP visualization of scGT colored by cell types. Red circle shows the location of Myeloid cells and purple circle shows the location of Cardiomyocyte cells. **b** The confidence scores for scGT in the UMAP visualization of snATAC-seq data. Red circle shows the location of Myeloid cells and purple circle shows the location of Cardiomyocyte cells. **c** Violin plots of confidence scores for scGT categorized by cell types. **d** Heatmap of corrected and normalized label transfer confusion matrix for scGT, taking a threshold of 0.9. Cells with a confidence score less than 0.9 are classified as Unknown type.

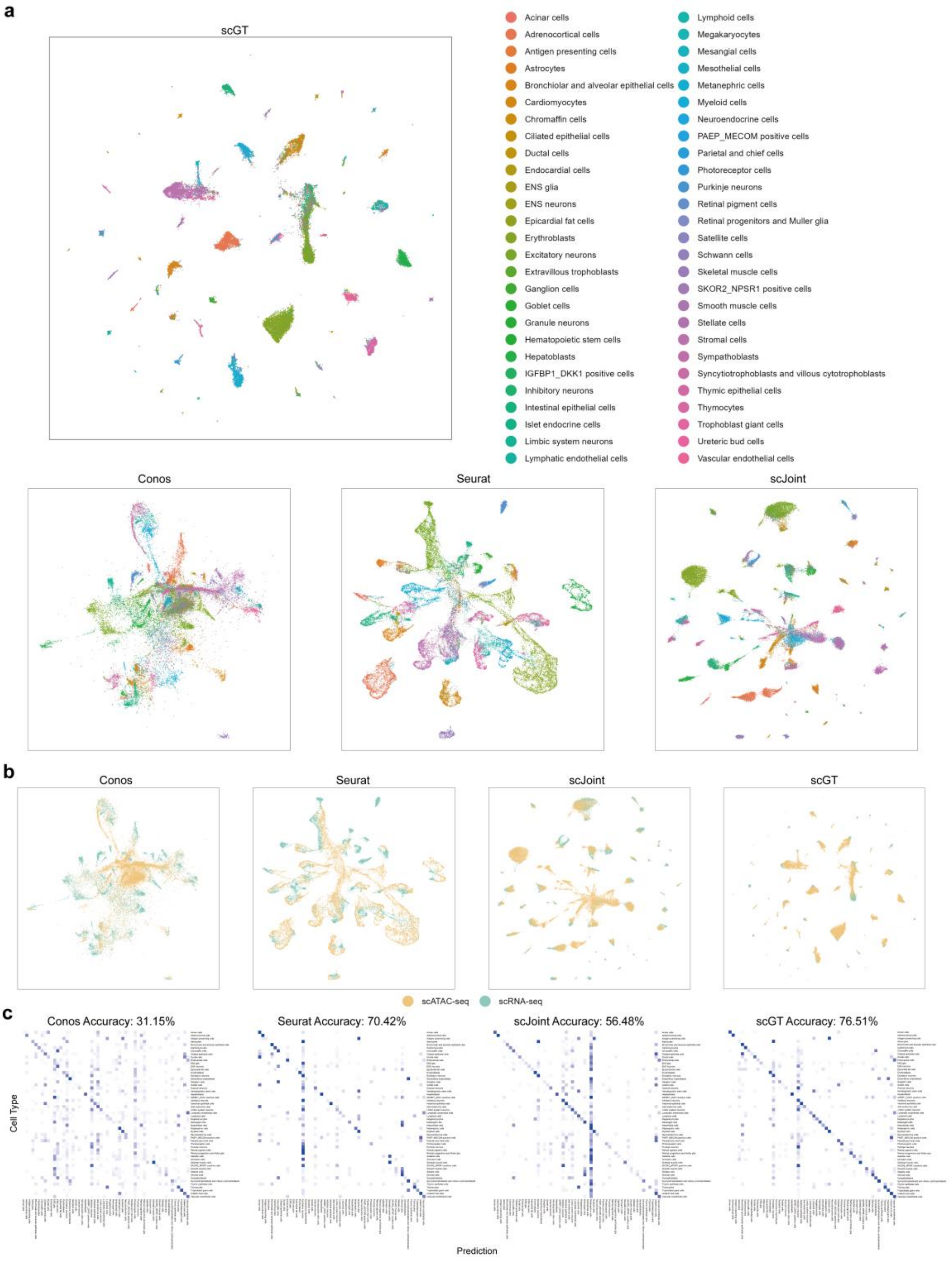

Supplementary Fig. 4 Human fetal atlas 40,000 subset. a UMAP visualization of four methods on human fetal atlas 40,000 subset data colored by cell types. **b** UMAP visualization of four methods on human fetal atlas 40,000 subset data colored by omics. **c** Heatmap of normalized label transfer confusion matrix for four methods.

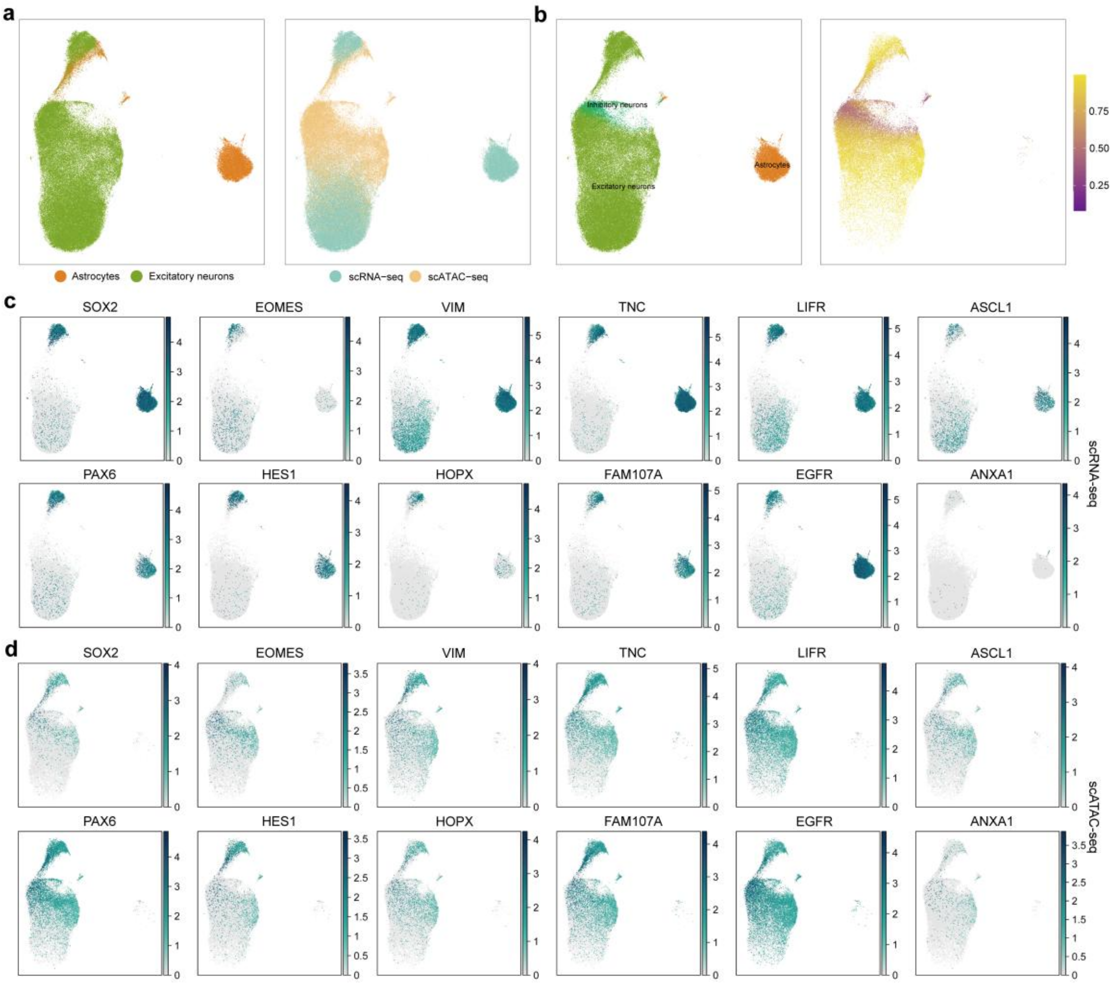

Supplementary Fig. 5 Analysis of Astrocytes and Excitatory neurons on human fetal atlas data. **a** UMAP visualization of scGT's embedding on Astrocytes and Excitatory neurons, colored by cell types (left) and omics (right). **b** UMAP visualization of scGT's embedding on Astrocytes and Excitatory neurons, colored by predicted type (left). The confidence scores for scGT in the UMAP visualization of scATAC-seq data (right). **c** Expression of 12 marker genes in scRNA-seq data. **d** Expression of 12 marker genes in scATAC-seq data.

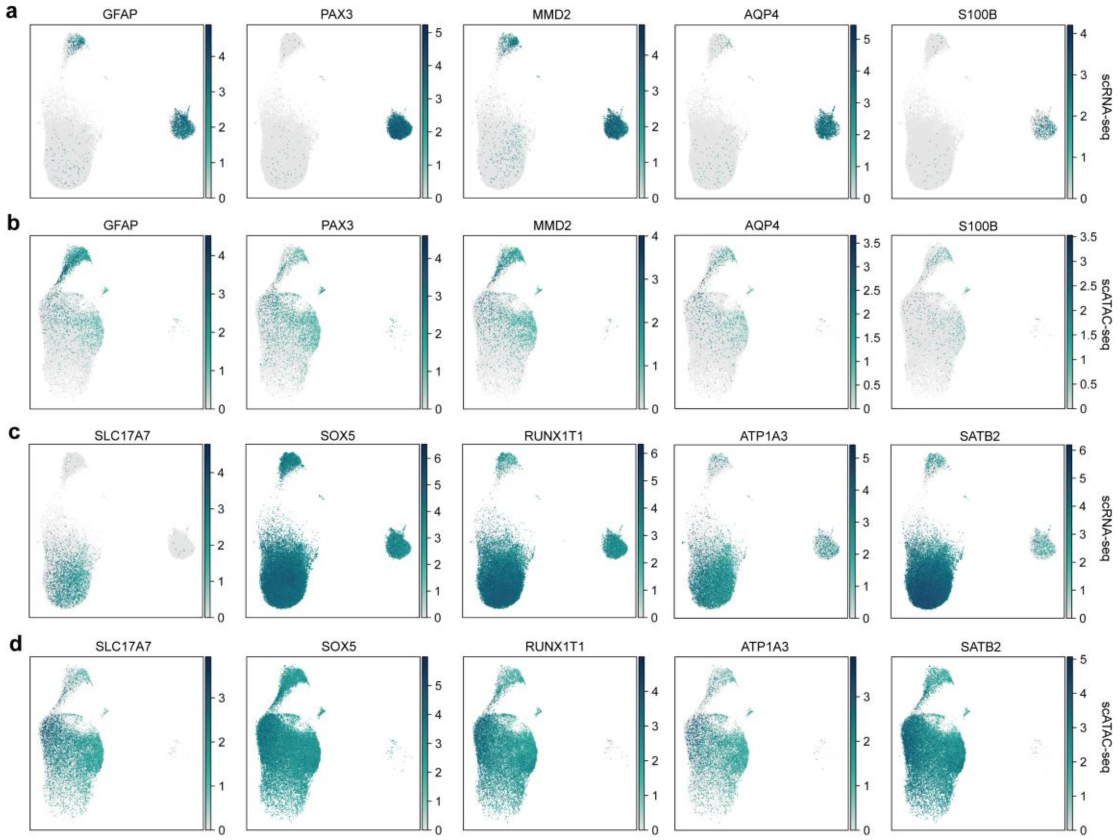

Supplementary Fig. 6 Supplementary analysis of Astrocytes and Excitatory neurons on human fetal atlas data. a Expression of 5 marker genes of Astrocytes from scRNA-seq data. **b** Expression of 5 marker genes of Astrocytes from scATAC-seq data. **c** Expression of 5 marker genes of Excitatory neurons from scRNA-seq data. **d** Expression of 5 marker genes of Excitatory neurons from scATAC-seq data.

Supplementary Note

A: Data availability and preprocessing

scGT accepts the raw gene expression matrix and gene activity matrix as input. Prior to integration, scGT will by default divide the read counts of each cell in the scRNA-seq data by the total read counts across all genes, then multiply by 10,000, followed by log transformation and scaling the data to unit variance and zero mean. For the gene activity matrix of scATAC-seq, we apply TFIDF transformation and scale the data to unit variance and zero mean (Li *et al.* 2023). The preprocessing for each dataset is as follows.

- Mouse colon data (Ma *et al.* 2020). The gene expression matrix, peak-by-cell matrix, fragments file, and cell-type annotations were downloaded from GSE207308. The gene activity matrix was calculated using the R package ArchR v1.0.2 (Granja *et al.* 2021). We selected common cells, resulting in a total of 5,210 cells, with 16,695 common genes between the scRNA-seq and scATAC-seq data.
- Mouse brain cortex data (Chen *et al.* 2019). The gene expression matrix, peak-by-cell matrix were downloaded from GSE126074. The fastq downloaded from SRP183521. It was aligned with the mouse reference genome GRCm38 using bwa (Li and Durbin 2009), v0.7.17-r1188. Next, the fragment files were generated from the alignment using Sinto v0.10.1. Additionally, the gene activity matrix was generated using R package ArchR v1.0.2, resulting in a dataset with 8815 cells and 18738 genes for analysis.
- Human myocardial infarction data (Kuppe *et al.* 2022). The gene expression matrix, gene activity matrix, and cell-type annotations were downloaded from https://cellxgene.cziscience.com/collections/8191c283-0816-424b-9b61-c3e1d6258a77. This dataset includes 191,795 snRNA-seq cells and 46,086 snATAC-seq cells. The peak-by-cell matrix were obtained from https://zenodo.org/record/6578553 and https://zenodo.org/record/6578617, and processed using the R package ArchR v1.0.2 with default parameters. To construct the type-mismatched dataset, we removed Myeloid cells from the snRNA-seq data. Additionally, to construct a balanced dataset, we sampled cell types in the snRNA-seq data with more than 7,000 cells, with the sampling size set to $\max\{0.05n, 7000\}$, resulting in a total of 42,458 snRNA-seq cells. The two omics contain 17,865 common genes.
- Human peripheral blood mononuclear cells (PBMC) ((Mimitou *et al.* 2021)). The gene expression matrix and gene activity matrix were downloaded from https://github.com/SydneyBioX/scJoint/blob/main/data.zip. We removed the "unknown" and "DC" cells, resulting in a total of 4,644 CITE-seq cells and 4,157 ASAP-seq cells. For all methods, we only used gene expression matrix and gene activity matrix. Ultimately, the two omics contain 14,303 common genes. Fragment files were downloaded from GSE156478 and then computed to obtain the peak-by-cell matrix using the R package ArchR v1.0.2, where we used the default parameters of ArchR.

- Human fetal atlas data (Cao et al. 2020; Domcke et al. 2020). The gene expression matrix and cell-type annotations for the scRNA-seq data were downloaded from GSE156793, while the gene activity matrix and peak-by-cell matrix for the scATAC-seq data were obtained from GSE149683. We selected 54 cell types common to both atlas datasets. For cell types in the scRNA-seq data with more than 10,000 cells, with the sampling size set to max{0.05n, 10,000}, resulting in a total of 433,695 cells. The scATAC-seq data contains 656,074 cells.

B: Ablation study

We tested the effect of the scGT algorithm using raw graph and filtered graph respectively. We ran three rounds of experiments and calculated the corresponding metrics. It shows that the label transfer accuracy as well as all the metrics are improved to different degrees after using filtered graph.

Supplementary Table S1. Ablation study on mouse colon data

		Accuracy	F1 Silhouette coefficient (mean)	ASW	MAP
scGT with filtered graph	Run1	74.78%	0.5630	0.7113	0.8232
	Run2	76.03%	0.5682	0.7272	0.8223
	Run3	75.37%	0.5668	0.7209	0.8263
	**Mean**	**75.39%**	**0.5660**	**0.7198**	**0.8239**
scGT with raw graph	Run1	72.44%	0.5606	0.7063	0.8107
	Run2	72.61%	0.5615	0.6984	0.8065
	Run3	72.92%	0.5619	0.7020	0.8114
	**Mean**	**72.66%**	**0.5613**	**0.7022**	**0.8095**

Supplementary Table S2. Ablation study on mouse brain cortex data

		Accuracy	F1 Silhouette coefficient (mean)	ASW	MAP
scGT with filtered graph	Run1	73.05%	0.5619	0.7052	0.8087
	Run2	73.77%	0.5616	0.7159	0.8076
	Run3	73.82%	0.5619	0.7129	0.8072
	**Mean**	**73.55%**	**0.5618**	**0.7113**	**0.8078**
scGT with raw graph	Run1	72.84%	0.5540	0.6781	0.8025
	Run2	73.84%	0.5581	0.6888	0.8034
	Run3	73.33%	0.5621	0.6998	0.8022
	**Mean**	**73.34%**	**0.5581**	**0.6889**	**0.8027**

Supplementary Table S3. Ablation study on human peripheral blood mononuclear cells

		Accuracy	F1 Silhouette coefficient (mean)	ASW	MAP
scGT with filtered graph	Run1	84.65%	0.6085	0.8488	0.8936
	Run2	84.32%	0.6053	0.8474	0.8926
	Run3	84.44%	0.6064	0.8452	0.8921
	**Mean**	**84.47%**	**0.6067**	**0.8471**	**0.8928**
scGT with raw graph	Run1	82.73%	0.5935	0.8067	0.8902
	Run2	80.59%	0.5924	0.8082	0.8782
	Run3	81.93%	0.5996	0.8237	0.8841
	**Mean**	**81.75%**	**0.5952**	**0.8128**	**0.8841**

Supplementary Table S4. Ablation study on 40k human fetal atlas data

		Accuracy	F1 Silhouette coefficient (mean)	ASW	MAP
scGT with filtered graph	Run1	76.51%	0.5240	0.6680	0.8370
	Run2	76.96%	0.5308	0.6973	0.8409
	Run3	76.35%	0.5324	0.6972	0.8352
	**Mean**	**76.61%**	**0.5291**	**0.6875**	**0.8377**
scGT with raw graph	Run1	73.65%	0.5210	0.6475	0.8343
	Run2	73.75%	0.5336	0.6751	0.8369
	Run3	72.67%	0.5248	0.6623	0.8300
	**Mean**	**73.36%**	**0.5265**	**0.6616**	**0.8337**

Reference

Cao,J. *et al.* (2020) A human cell atlas of fetal gene expression. *Science*, **370**, eaba7721.

Chen,S. *et al.* (2019) High-throughput sequencing of the transcriptome and chromatin accessibility in the same cell. *Nat. Biotechnol.*, **37**, 1452–1457.

Domcke,S. *et al.* (2020) A human cell atlas of fetal chromatin accessibility. *Science*, **370**, eaba7612.

Granja,J.M. *et al.* (2021) ArchR is a scalable software package for integrative single-cell chromatin accessibility analysis. *Nat. Genet.*, **53**, 403–411.

Kuppe,C. *et al.* (2022) Spatial multi-omic map of human myocardial infarction. *Nature*, **608**, 766–777.

Li,H. and Durbin,R. (2009) Fast and accurate short read alignment with Burrows-Wheeler transform. *Bioinformatics*, **25**, 1754–1760.

Li,Y. *et al.* (2023) scBridge embraces cell heterogeneity in single-cell RNA-seq and ATAC-seq data integration. *Nat. Commun.*, **14**, 6045.

Ma,S. *et al.* (2020) Chromatin Potential Identified by Shared Single-Cell Profiling of RNA and Chromatin. *Cell*, **183**, 1103-1116.e20.

Mimitou,E.P. *et al.* (2021) Scalable, multimodal profiling of chromatin accessibility, gene expression and protein levels in single cells. *Nat. Biotechnol.*, **39**, 1246–1258.
